# Evaluation of community health worker's performance at home-based newborn assessment supported by mHealth in rural Bangladesh

**DOI:** 10.1186/s12887-022-03282-6

**Published:** 2022-04-22

**Authors:** Farjana Jahan, Eric Foote, Mahbubur Rahman, Abul Kasham Shoab, Sarker Masud Parvez, Mizanul Islam Nasim, Rezaul Hasan, Shams El Arifeen, Sk Masum Billah, Supta Sarker, Md. Mahbubul Hoque, Mohammad Shahidullah, Muhammad Shariful Islam, Sabina Ashrafee, Gary L. Darmstadt

**Affiliations:** 1 Environmental Intervention Unit, International Centre for Diarrheal Disease Research, Bangladesh (icddr,b), Dhaka, Bangladesh; 2 Department of Pediatrics, Stanford University School of Medicine, Stanford, CA USA; 3 Faculty of Medicine, The University of Queensland, Brisbane, QLD Australia; 4 Maternal and Child Health Division, International Centre for Diarrheal Disease Research, Bangladesh (icddr,b), Dhaka, Bangladesh; 5 Faculty of Medicine and Health, Sydney School of Public Health, The University of Sydney, Sydney, Australia; 6 Department of Neonatology, Dhaka Shishu (Children) Hospital, Dhaka, Bangladesh; 7 Bangabandhu Sheikh Mujib Medical University, Dhaka, Bangladesh; 8 National Newborn Health Program (NNHP) and Integrated Management of Childhood Illness (IMCI), Directorate General of Health Services, Dhaka, Bangladesh

**Keywords:** mHealth, Community health workers, Neonatal health, Neonatal jaundice

## Abstract

**Background:**

In low to middle-income countries where home births are common and neonatal postnatal care is limited, community health worker (CHW) home visits can extend the capability of health systems to reach vulnerable newborns in the postnatal period. CHW assessment of newborn danger signs supported by mHealth have the potential to improve the quality of danger sign assessments and reduce CHW training requirements. We aim to estimate the validity (sensitivity, specificity, positive and negative predictive value) of CHW assessment of newborn infants aided by mHealth compared to physician assessment.

**Methods:**

In this prospective study, ten CHWs received five days of theoretical and hands-on training on the physical assessment of newborns including ten danger signs. CHWs assessed 273 newborn infants for danger signs within 48 h of birth and then consecutively for three days. A physician repeated 20% (*n* = 148) of the assessments conducted by CHWs. Both CHWs and the physician evaluated newborns for ten danger signs and decided on referral. We used the physician’s danger sign identification and referral decision as the gold standard to validate CHWs’ identification of danger signs and referral decisions.

**Results:**

The referrals made by the CHWs had high sensitivity (93.3%), specificity (96.2%), and almost perfect agreement (K = 0.80) with the referrals made by the physician. CHW identification of all the danger signs except hypothermia showed moderate to high sensitivity (66.7–100%) compared to physician assessments. All the danger signs assessments except hypothermia showed moderate to high positive predictive value (PPV) (50–100%) and excellent negative predictive value (NPV) (99–100%). Specificity was high (99–100%) for all ten danger signs.

**Conclusion:**

CHW's identification of neonatal danger signs aided by mHealth showed moderate to high validity in comparison to physician assessments. mHealth platforms may reduce CHW training requirements and while maintaining quality CHW physical assessment performance extending the ability of health systems to provide neonatal postnatal care in low-resource communities.

**Trial registration:**

clinicaltrials.gov NCT03933423, January 05, 2019.

## Introduction

Neonatal mortality remains a major challenge for achieving Sustainable Development Goal (SDG) 3 for good health and well-being in low and lower-middle-income countries (LMICs) [1]. Over the 20 years, Bangladesh made progress in reducing under-5 mortality especially the post neonatal and child mortality [2]. But newborn mortality still remains a big challenge and contributes 61% of all under-5 mortality. Three-quarters of neonatal deaths occur in the first week of life and nearly half occur in the first 24 h, of which more than half occur at home [2–5].

In Bangladesh, despite a reduction in neonatal mortality rate from 52 per 1000 live births in 1993–1994 to 30 per 1000 live births in 2017–18, the decline is slow, and the rate remains well above the 12 per 1000 live birth target of SDG 3 by 2030 [6, 7]. Approximately 50% of mothers in Bangladesh deliver at home, and a medical provider sees 52% of women and children within 48 h of delivery. However, only 7% of home births are seen by a medical provider within 48 h of birth [8]. To improve health and survival in the first month after birth, community-based interventions to link the community and healthcare system using home visits by CHWs have been explored extensively in LMIC settings [9–17]. CHW neonatal postnatal home visits have reduced neonatal mortality by about 30–60% among communities [17–19].

During postnatal home visits, trained CHWs perform physical assessments to screen newborns for danger signs and those newborns with danger signs are referred to a healthcare center for treatment [20–22]. In order for CHWs to be able to make quality neonatal danger sign assessments, resource intensive trainings with a long duration were required varying from 10 days to 36 days, limiting countrywide scale up of CHW-led newborn postnatal programs [14, 23, 24].

Mobile applications and devices for public health activities (mHealth) have been explored to aid CHWs to provide maternal and child care in different settings across the world [25–29]. These tools can support in training, communication, data collection, decision-making, appropriate supervision, monitoring, management, and improve the performance and motivation of the CHWs [30, 31]. In different settings, mHealth based programs modeled on the identification of mother and newborn at risk, delivery of care and follow-up demonstrated increases in outreach and feasibility [32–35]. In Bangladesh, a number of studies evaluated and demonstrated potential applications of mHealth in engaging CHWs in delivering maternal and newborn health intervention including message delivery for health promotion, identification of sick newborn and emergency referral [36–40].

In this study, trained CHWs were evaluated for their ability to identify newborn danger signs during postnatal visits with the help of an mHealth tool with a built-in clinical algorithm.

We aimed to evaluate the effectiveness of our CHW training program to train CHWs to perform quality newborn danger sign assessments with the aid of mHealth in comparison to CHW training programs with longer training duration with a shorter training period and comprehensive training on improving CHWs’ assessment of neonatal danger signs and the need for referral compared to physician assessment as the gold standard.

## Methods

### Study design and population

This prospective study was conducted within a cluster-randomized controlled intervention trial of home-based management of neonatal jaundice in Sakhipur Upazilla, Tangail, Bangladesh from September 2019 to March 2020 and October 2020 to April 2021. Sakhipur subdistrict of Dhaka division, Bangladesh is a rural agrarian community with a population of ~ 300,000 people in 132 villages, approximately 80 km from the capital city of Dhaka. A government hospital with 50 beds provides caesarean sections, vaginal birthing services, and emergency services. Poverty, crowding, unstable housing, food insecurity, and poor hygiene and sanitation are common [41]. About 82% of women in Dhaka division receive at least one antenatal care (ANC) visit from a trained medical provider, and 73% of women receive quality antenatal care [8]. Approximately 50% of mothers in the selected division (Dhaka) deliver at home, and a medical provider sees 52% of women and children within 48 h of delivery. However, only 7% of home births are seen by a medical provider within 48 h of birth [8]. If a neonatal hyperbilirubinemia case is diagnosed in Sakhipur and requires phototherapy, newborn infants are referred to a hospital that takes at least 2 h to reach [42].

For participant recruitment, we completed a listing of 650 pregnant mothers within 14–26 weeks of pregnancy from the Shakhipur sub-district along with Global Positioning System (GPS) locations of the households. We then formed 20 clusters, each comprised of 25–29 pregnant mothers residing in the closest geographical proximity. We eliminated households that did not form a cluster according to geographical proximity. We used Excel’s rand function to assign clusters to intervention or control randomly. Randomization resulted in 10 clusters in each arm. All the live newborns of the enrolled pregnant mothers in the intervention clusters were included in the study for danger signs assessment and were assessed within 48 h of birth.

### Recruitment of the CHWs

Ten CHWs were recruited through a competitive recruitment process at the beginning of the project from the local community. We assigned one CHW to each of the ten intervention clusters. All the CHWs were female, ranging from 22 to 40 years of age and had a minimum of higher secondary education. A few of them had prior experience working in the community, and most of them had little knowledge of pregnant mother and newborn health care.

### Training of the CHWs

We developed a training manual on prenatal educational sessions for mothers on antenatal care, neonatal jaundice, and home-based screening and management of neonatal jaundice in English and Bangla by adapting existing materials used in similar programs and standardizing the contents through face and content validation. All CHWs were given 5 days of training in two phases: perinatal care of the mother and management of neonatal jaundice (Table 1). Training included 3 days of theoretical and practical training at Sakhipur Upazilla Health Complex (UHC) and 2 days of bedside hands-on training at Dhaka Children Hospital; a tertiary level specialized hospital in Dhaka, Bangladesh. The research team evaluated their CHWs’ pretest and posttest performance using a structured assessment tool. In addition, during hands-on training, the trainers observed each CHW assessing five newborns in the neonatal ward for danger signs. We conducted refresher training monthly. A weekly meeting was organized for experience sharing and review of study progress.

**Table 1:** Curriculum contents of the community health worker training

Theoretical and practical training topics	The scenario of maternal and child health in Bangladesh, neonatal jaundice, and responsibilities of community health workers
	Pregnancy care and maternal danger signs
	Delivery planning and safe delivery preparations
	Newborn care
	Prevention of maternal and neonatal infections
	Breastfeeding and management of breastfeeding problems
	Recognizing neonatal illness and common problems
	Physical examination and evaluation of the newborn after birth
	Postpartum services and the responsibilities of community health workers
	Referral system of sick newborn and its components
	Neonatal jaundice, types, and symptoms
	Risk of developing neonatal jaundice and management
	Management of neonatal jaundice in the community
	Description of equipment and materials associated with neonatal jaundice management
	Communication and counseling to caregivers
	Using an electronic handheld tablet and CommCare for case management
Bedside hands-on training topics	General examination of a neonate
	Danger signs of the neonate
	Infection prevention of the newborn
	Neonatal jaundice signs, symptoms, and management
	Breastfeeding and management of breastfeeding problems

### Study procedure

**Fig. 1:**
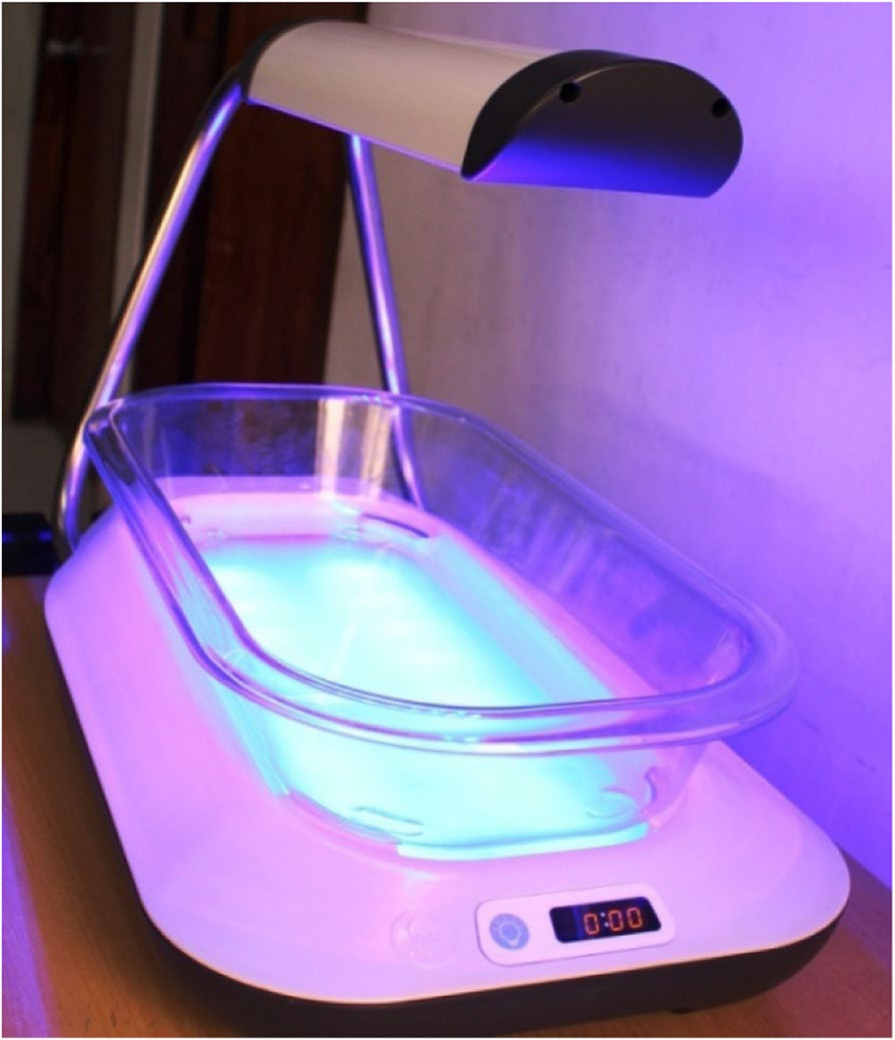
Image of Firefly® phototherapy system (MTTS Asia, Hanoi Vietnam) used in the study (source: icddr,b)

The CHWs visited each newborn within 48 h of birth and then daily for three consecutive days to assess for hyperbilirubinemia and to evaluate breastfeeding and institute phototherapy if indicated. Total bilirubin was measured using a transcutaneous bilimeter (Draeger JM-105). During CHWs visits, if any danger signs was observed, CHWs referred the newborns to an appropriate medical facility. Newborns eligible for home treatment were treated with LED phototherapy using Firefly® phototherapy system (MTTS Asia, Hanoi Vietnam), a portable double surface phototherapy device, for 36 h. During the phototherapy period, CHWs assessed newborns daily for danger signs (Fig. 1) [43].

A designated physician repeated the same assessments of newborn danger signs independently on the same day as the CHW assessment within 4 h. The designated physician repeated 20% of the total assessments conducted by each CHW. On each day, the physician collected the list of the households the CHWs visited and conveniently picked households to repeat assessments. Physicians visited the households and repeated the CHWs assessments in newborns, both with and without danger signs. Physicians’ identification of danger signs was used as the gold standard in determining the validity (sensitivity, specificity, and positive and negative predictive values) of CHWs’ identification of danger signs of illness. For referral, the physician’s decision on referral was considered as the final decision to prevent unnecessary referral.

### mHealth case management and decision support

**Fig. 2:**
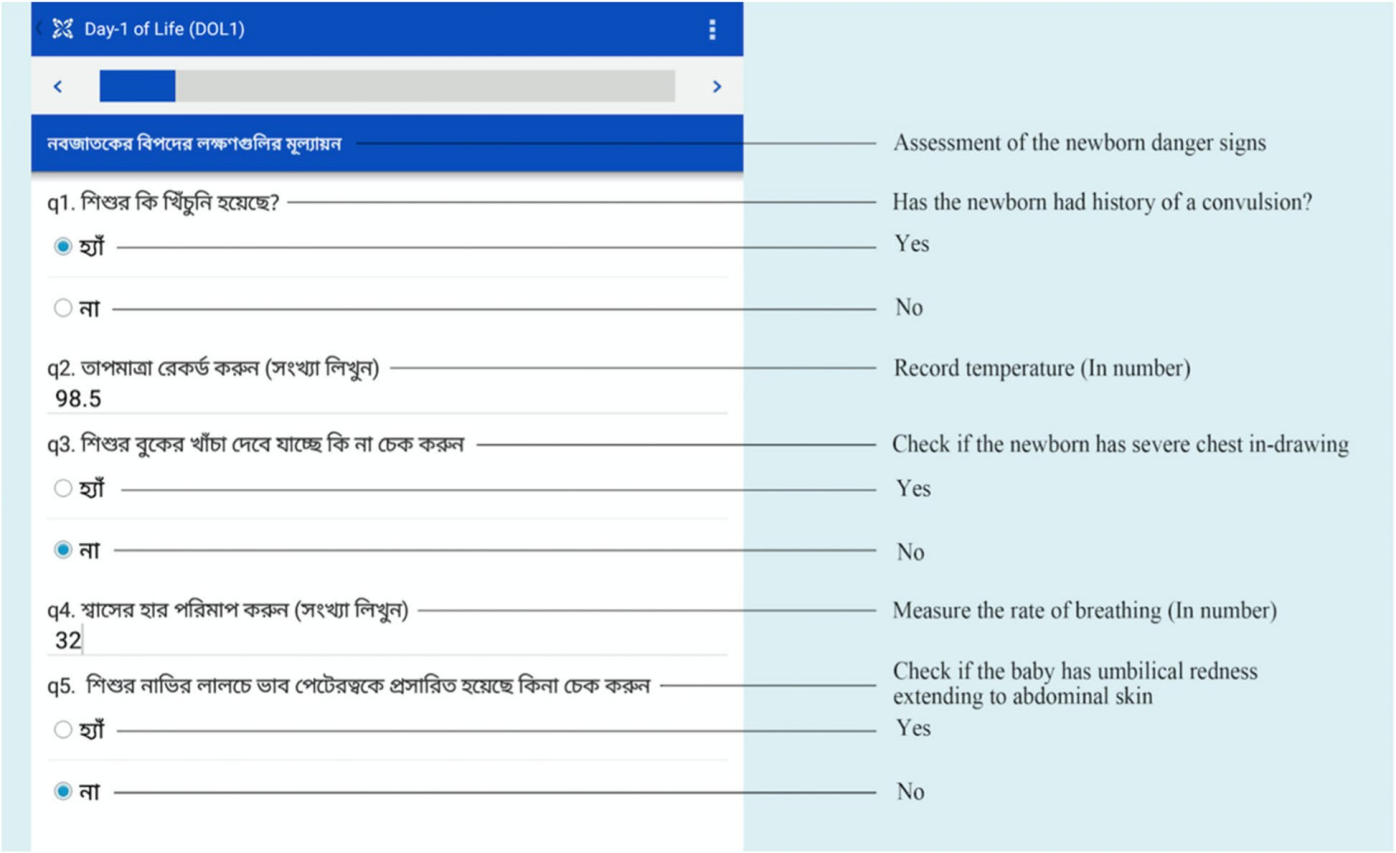
Illustration of the mHealth CommCare platform with newborn danger sign assessment (Source: icddr,b)

We developed an mHealth application (Fig. 2) for CHWs using CommCare software program (Dimagi, Cambridge, USA) on an electronic handheld tablet to guide CHWs through newborn danger signs assessment. Successful use of the CommCare based mHealth applications by CHWs has been demonstrated previously [44]. The application contained pertinent information on the participants, including location, gestational age, blood group, and Rh type of pregnant women and their husbands.

The application developed on CommCare calculated an estimated delivery date based on the first day of the last menstrual period [45]. Gestational age was calculated by subtracting the birth date from the first day of the last menstrual period. The birth weight of the newborn was directly measured and also obtained by reviewing delivery records from the hospital. Birth weight of the newborns were measured by Seca weight scale (model: 3831317009). Age of the baby in hours, was determined based on birth time reported by the mother or from records among hospital births. The mobile tablet guided the CHWs in 1) danger sign assessment of the newborn and mother, 2) breastfeeding assessment, and 3) neonatal jaundice case management by an algorithm based on AAP guidelines [46]. In every referral decision, the program provided a brief note describing the problem and emphasized the need for the referral for the CHWs and family members to understand. CHWs then facilitated the referral to Sakhipur sub-district hospital with the help of family members and the physician. CHWs communicated with the referral centre to ensure the referred newborn had been managed in the referral centre.

The application had data quality checks (answers constraints and requires questions to be answered before advancing) to improve the accuracy of CHW recommendations.

The neonatal danger signs used in this study were adopted from the Neonatal National Health Strategy which were validated previously in Bangladesh [23, 47, 48]. The algorithm included ten danger signs for assessment. If a danger sign was present, the program prompted for referral. We added no void in 24 h to the danger signs because we were going to be treating babies with phototherapy and did not want to treat babies who were already dehydrated. In case of hypothermia, CHWs advised mothers to warm up the babies and provide kangaroo mother care. After 30 min of warm-up, if the baby was still hypothermic, they referred the baby. If any of the following danger signs were present or if the newborn was < 2000 g or < 35 weeks gestational age at birth, the newborn was referred to nearest UHC for treatment.

1. History of Convulsions- CHWs asked mother if the baby had a convulsion after birth
2. Hypothermia- if the axillary temperature measured by a digital thermometer was less than 35.5 °C or 95.9 °F
3. Fever- if the axillary temperature measured by a digital thermometer was more than 37.5 °C or 99.5 °F
4. Severe chest in drawing- CHWs checked if the newborn had severe chest indrawing
5. Tachypnea (> 60 breath/min on two consecutive readings, 30 min apart)
6. Umbilical redness extending to abdominal skin- CHWs checked if the baby has umbilical redness extending to abdominal skin
7. Movement only when stimulated or no movement at all-
8. No void within 24 h- CHWs asked the mother if the baby had no void in last 24 h
9. Jaundice of palms or soles- CHWs checked if the yellowness of body extended to palm or soles
10. Feeding difficulty- CHWs asked the mother if the baby had any feeding difficulty

### Statistical analysis

The CHW–physician assessment pair was the unit of analysis. We examined the association between the assessment by physicians and assessment by the CHW to identify individual danger signs. Sensitivity, specificity, positive predictive value (PPV), and negative predictive value (NPV) were calculated considering the assessment by the physician as the gold standard. Kappa statistics (K) were calculated for determining agreement between physicians and CHWs. K < 0 was considered poor, K = 0.0–0.20 slight, K = 0.21–0.40 fair, K = 0.41–0.60 moderate, K = 0.61–0.80 substantial and K = 0.81–1.00 was considered almost perfect agreement [49]. Kappa tests were done considering alpha< 0.05 as statistically significant. STATA 16 software package (Stata Corporation, College Station, TX, United States of America) was used for all the analysis.

### Ethical approval and consent to participate

We took written informed consent from all the participants. Field researchers explained study objectives, activities, anonymity, and confidentiality to the participants and assured them that they could withdraw anytime. Any modifications to the protocol was communicated to the Institutional review boards to be approved for ethical clearance. All methods were performed in accordance to the trial protocol and guidelines. This study was approved by the Research Review Committee and the Ethical Review Committee of icddr,b, protocol # 19004 and the Stanford University Institutional Review Board. The trial was registered at clinicaltrials.gov (NCT03933423) on 01/05/2019.

#### Safety review plan

An independent data safety monitoring committee (DSMB) was assembled in Bangladesh to monitor adverse events and to advise investigators with five respective members. The board was composed of two neonatologists, two epidemiologists, and one demographer. Stanford University institutional review board also reviewed the protocol prior to beginning the study. Any cases of acute bilirubin encephalopathy, exchange transfusion, or death during the trial was considered a serious adverse event. The investigators reported serious adverse events to the Data Safety Monitoring Board within 24 h of the event. The Data Safety Monitoring Board was responsible for evaluating the event and determining the next steps. To ensure project safety, study staff and a project research physician were always present in the field site.

## Results

### Characteristics of CHWs

The mean age of the CHWs was 28.1 (±3.2) years. About three quarters (71%) of the CHWs had completed graduation, and no CHWs had any previous training on identifying newborn danger signs.

### Assessments

A total of 273 newborns born between November 2019 to March 2020 and October 2020 to May 2021 were assessed by the CHWs. CHWs completed a total of 740 assessments. Among 273 newborns, 27 (9.8%) newborns were referred or started phototherapy on the first day of assessment, and 28 (10.2%) were referred or started phototherapy on the second day of assessment.

### Characteristics of the newborns

Out of the 148 newborns assessed by both the CHWs and the physician, 45% were female (Table 2). Eight percent of the neonates were born before 35 weeks gestational age, 16% were born between 35 and 37 weeks of gestation, and 41% were born at home. The mean birth weight was 2658 (±375) grams and only nine (6%) of the newborns had low birth weight (< 2500 g) (Table 2). There were no babies with birth weight less than 2000 g. The average hour of life of the newborns during first assessments was 50 (SD 39) hours.

**Table 2:** Characteristics of newborn infants included in the CHW assessment validation study in Shakhipur, Bangladesh (2019–2020)

Characteristics	Frequency (*n* = 148)	Percentage (%)
Sex		
Female	67/148	45
Male	81/148	55
Gestational age		
< 35 weeks	11/148	8
35–37 weeks	25/148	16
≥ 37 weeks	112/148	76
Birth place		
UHC	9/148	6
District/ Medical college hospital	5/148	3
Private hospital	71/148	48
NGO clinic	2/148	1
Home	61/148	41
Mode of delivery		
Normal vaginal delivery	85/148	57
Cesarean section	63/148	43
Birth weight (in grams), mean (SD)	2658 (375)	
Birthweight		
≥ 2500 g	139	94
2000–2499 g	9	6
Hour of life during assessments	(Mean, SD)	
Assessment 1	50 ± 39	
Assessment 2	63 ± 30	
Assessment 3	118 ± 51	

### Assessment of newborn danger signs

During 148 CHWs’ assessments, 16 (11%) newborns had a single danger sign, one had two danger signs, and two newborns had four danger signs. The danger signs found most frequently during CHW assessments were no void in 24 h (4.7%), newborns with yellow palms and soles (2.7%), and difficulty feeding (2%) (Table 3). Hypothermia and hyperthermia were reported among 2 and 1.4% of newborns, respectively. Convulsions were found in 1.4% of newborns, and they moved only while being stimulated. Severe chest in-drawing and umbilical redness extending to the abdominal skin were observed in 0.7% of newborns. CHWs did not report any case of tachypnea, and sensitivity and specificity of tachypnea could not be reported.

**Table 3:** Distribution of danger signs by gestational age and birthweight of newborn

Danger signs	Total Frequency n (%)	Gestational age n (%)			Birth weight n (%)	
		< 35 weeks n (11)	35–37 weeks n (25)	> 37 weeks n (112)	2000–2499 g n (9)	> = 2500 g n (139)
Convulsion	2 (1.4)	0 (0)	0 (0)	2 (100)	0 (0)	2 (100)
Hypothermia	3 (2.0)	1 (33.3)	1 (33.3)	1 (33.3)	0 (0)	3 (100)
Hyperthermia	2 (1.4)	0 (0)	0 (0)	2 (100)	0 (0)	2 (100)
Severe chest-indrawing	1 (0.7)	0 (0)	0 (0)	1 (100)	0 (0)	1 (100)
Tachypnea	0 (0)	0 (0)	0 (0)	0 (0)	0 (0)	0 (0)
Umbilical redness extending to the skin	1 (0.7)	1 (100)	0 (0)	0 (0)	0 (0)	1 (100)
Only moves when stimulated	2 (1.4)	1 (50)	0 (0)	1 (50)	0 (0)	2 (100)
No void in 24 h	7 (4.7)	2 (29)	1 (14)	4 (57)	0 (0)	7 (100)
Yellow palm or soles	4 (2.7)	0 (0)	0 (0)	4 (100)	0 (0)	4 (100)
Feeding difficulty	3 (2.0)	1 (33)	0 (0)	2 (67)	0 (0)	3 (100)

Convulsion, severe chest in drawing, umbilical redness extending to abdominal skin and no void in 24 h had 100% sensitivity and almost perfect agreement (K = 0.83–1.00). Feeding difficulty and no movement or only moves when stimulated had moderate to high sensitivity and positive predictive value resulting in almost perfect agreement (K = 0.85, 0.83). Hyperthermia and yellow palm or soles had 100 and 75% sensitivity while positive predictive values were 50 and 75%, respectively resulting in substantial agreement (K = 0.66, 0.74). On the other hand, hypothermia had 50% sensitivity and 33.3% positive predictive value, and the agreement was fair (K = 0.39). Specificity and negative predictive value were above 99% for all the danger signs (Table 4).

**Table 4:** Frequency (%) and validity of clinical signs observed, referral decision and Kappa statistic for agreement between assessments by CHWs and physician gold standard

Newborn danger signs assessed	Number of children had the sign/symptoms (identified by physician) n (%)	Number of children did not have the sign/symptoms (identified by physician) n(%)	True negative	False negative	False positive	True positive	Sensitivity % (CI)	Specificity % (CI)	PPV % (CI)	NPV % (CI)	K	*p*-value
Convulsion (*N* = 148)	2 (1.4)	146 (98.7)	146	0	0	2	100.0 (15.8–100)	100.0 (97.5–100)	100.0 (15.8–100)	100.0 (97.5–100)	1.00	0.000
Hypothermia (*N* = 148)	1 (0.7)	147 (99.3)	144	1	2	1	50.0 (1.3–98.7)	98.6 (95.1–99.8)	33.3 (0.8–90.6)	99.3 (96.2–100)	0.39	0.000
Hyperthermia (*N* = 148)	1 (0.7)	147 (99.3)	146	0	1	1	100 (2.5–100)	99.3 (96.3–100)	50.0 (1.3–98.7)	100 (97.5–100)	0.66	0.000
SevereFechest in-drawing (*N* = 148)	1 (0.7)	147 (99.3)	147	0	0	1	100 (2.5–100)	100 (97.5–100)	100 (2.5–100)	100 (97.5–100)	1.00	0.000
Tachypnea (*N* = 148)	1 (0.7)	147 (99.3)	147	0	1	0	(*) one-sided, 97.5% confidence interval					
Baby has umbilical redness extending to abdominal skin (*N* = 148)	1 (0.7)	147 (99.3)	147	0	0	1	100.0 (2.5–100)	100.0 (97.5–100)	100.0 (2.5–100)	100.0 (97.5–100)	1.00	0.000
Only moves when stimulated (*N* = 148)	3 (2)	145 (98)	145	1	0	2	66.7 (9.4–99.2)	100 (97.5–100)	100 (15.8–100)	99.3 (96.2–100)	0.80	0.000
Newborn had no void (urine) in 24 h (*N* = 148)	5 (3.4)	143 (96.6)	141	0	2	5	100.0 (47.8–100)	98.6 (95.0–99.8)	71.4 (29.0–96.3)	100.0 (97.4–100)	0.83	0.000
Newborn had yellow palm or soles of feet (*N* = 148)	4 (2.7)	144 (97.3)	143	1	1	3	75.0 (19.4–99.4)	99.3 (96.2–100)	75.0 (19.4–99.4)	99.3 (96.2–100)	0.74	0.000
Baby having difficulty feeding (*N* = 148)	4 (2.7)	144 (97.3)	144	1	0	3	75.0 (19.4–99.4)	100 (97.5–100)	75.0 (29.4–100)	99.3 (96.2–100)	0.85	0.000
CHW decision on assessment Baby has danger signs and need referral (*N* = 148)	15 (10.1)	133 (89.9)	128	1	5	14	93.3 (68.1–99.8)	96.2 (91.4–98.8)	73.7 (48.8–90.9)	99.2 (95.8–100)	0.80	0.000

(*) one-sided, 97.5% confidence interval

*SE* Standard Error, *TN* True Negative, *FN* False negative, *FP* False Positive, *TP* True Positive

Some validity measures could not be calculated for signs with no cases identified by CHW

According to the danger signs, 12.8% of the newborns were referred by CHWs. The sensitivity of referral was 93.3%, and specificity was 96.2%. Positive predictive value of referral was 73.7%, and negative predictive value was 99.2%. Agreement between CHW referral and physician referral was almost perfect (K = 0.80) (Table 4).

Physician’s assessment prevented five (25%) referrals that were made by CHWs. After being referred 15 (100%) of the newborns received management either at government hospital or private clinic. After referral, 13 (86.6%) newborns were admitted to the hospital, and two newborns were checked by pediatricians in a chamber.

## Discussion

This study was conducted to evaluate the validity of trained CHWs compared to a physician gold standard in assessing danger signs of newborns in a rural, low-resource setting in Bangladesh with the assistance of an algorithm run through a mobile device. Study results showed that, CHW led home visits led to 12.8% referral with a successful referral rate of 100%. This implies a high acceptance of CHWs based assessment at community level. However, this evaluation has its own limitation. Physician’s assessment might influence the success rate of referral, parents might be more convinced as the physician visited and assessed their newborn. Further research would be required to evaluate the acceptance of CHW’s referral if physician does not intervene. Similar to our findings, in other contexts mHealth based home visits have been shown to be effective in improving care seeking behavior in neonatal period [50–53]. Results showed that the referrals made by the CHWs had high sensitivity (93.3%), specificity (96.2%), and almost perfect agreement (K = 0.80) with the referrals made by the physician. Mobile health-based assessment showed high level of adequacy in identifying newborn with danger signs. This finding corresponds to the previous validation study conducted in Bangladesh [14, 22, 23].

The CHW training was relatively short compared to other studies (5 days, as opposed to 36 days of training in the Mirzapur study [22] and 42 days of training in the Sylhet study [14]. mHealth may have contributed to the ability of CHWs to perform quality danger sign assessments despite a shorter training program.

In a study conducted among 288 neonates in rural Sylhet, Bangladesh, CHWs identified and referred infants with danger signs categorized as very severe disease with a sensitivity of 91% (K = 0.847) and infants with possible very severe disease with a sensitivity of 81% (K = 0.783) compared to physician assessment [14]. The frequencies of both very severe disease and possible very severe disease were 25.7 and 20.5%, respectively, and there were equal numbers of well and sick newborns in the study. Another study conducted in Mirzapur, Bangladesh among 395 infants showed that CHWs identified infants with very severe disease and possible very severe disease with 72.7% (K = 0.63) and 33.3% (K = 0.33) sensitivity, respectively compared to physician gold standard [22]. In this study, very severe disease was defined, as presence of one or more signs among 11 danger signs: convulsions, unconscious, fast breathing, severe chest-indrawing, fever, hypothermia, severe skin pustules, umbilical redness extending to skin, weak, abnormal or absent cry, lethargic or less movement, and feeding difficulty [22]. Very severe disease was found among 2.8% of newborns, and possible very severe disease was found among 6.8% of the newborns. CHWs referred newborns identified with very severe disease or possible very severe disease in both studies. The frequency of referral in our current study was 12.8% while in these two studies, it was 9.6 and 26%, respectively.

Another study by Biswas et al. found that among babies 2–59 months of age, frontline community workers (Auxiliary Nurse Midwives, Anganwadi Workers) correctly assessed 33.6% of the children for all four danger signs following integrated management of neonatal and childhood illness (IMNCI) – which is lower than our study [54]. In a study conducted in Nepal among 1448 young infants, there was substantial agreement (K > 0.75) between home assessments conducted by female community health volunteers (FCHV) with basic training compared to home assessments conducted by highly trained facility-based community health workers (FBCHW) [24]. A recent study conducted in Ghana compared community volunteer assessments with non-physician trained supervisor observation and found high validity (79.5% sensitivity, 100% specificity, and 96.6% agreement) [21].

The positive predictive value (73.7%, 48.4–90.9% CI) of referral in our study indicates that about three-fourths of the newborns needing referral-level care were correctly identified. It also indicates that nearly one-fourth of the neonates were diagnosed inaccurately, which may have caused unnecessary referrals. However, subsequent physician assessment prevented five (25%) such referrals. NPV was high (99.2%, 95.8–100% CI) indicating there was less than 1 % chance of a sick newborn not being referred. These findings are on par with similar studies mentioned above.

All the danger signs assessments except hypothermia had moderate to high positive predictive value (50–100%) and excellent negative predictive value (98–100%). As this study was conducted in the general population, it implies CHWs’ assessment can identify and refer a good number of diseased newborns and simultaneously reduce unnecessary exposure of newborns to hospitals.

The low sensitivity and agreement of hypothermia can be a result of multiple limitations of this study. Once any newborn was found hypothermic, CHWs advised mothers to warm up the babies providing kangaroo mother care and warm clothes. The physician assessed these newborns after 4 h, and by this time, the babies became normothermic after warm-up procedures.

As the physician visited each household after the CHW visit, breathing rate may have changed by the time the physician visited the household (timing bias). Despite efforts to shorten the time gap between CHW visits and physician visits, a time lag is inevitable in this type of community-based study.

The prevalence of each of the danger signs in this study corresponded to other studies. Among the danger signs, no void in 24 h was the most prevalent (4.7%) and had high sensitivity (100%). Hyperthermia, convulsion, and umbilical redness had higher sensitivity (100%) than other validation studies (0–50%) [22]. Prevalence of yellow palm or soles was 2.7% with sensitivity of 75% which corresponds to the neonatal jaundice identification validation study in Karachi, Pakistan (83.3%) [55] but is higher than validation study in Bangladesh (21.4%) [14, 22, 23, 56]. The core study on neonatal jaundice, where CHWs measured the transcutaneous bilirubin to screen for neonatal jaundice may have influenced the CHWs ability to correctly identify this danger sign. Identification of this sign is also reliant on visual inspection and more objective than many other signs [57]. However, in a study conducted in Karachi, Pakistan, assessment of jaundice using visual inspection by primary health care workers had higher sensitivity than physicians’ inspection (83.3% vs 51.4%) but lower specificity (50.5% vs 90.7%) [55].

Feeding difficulty was one of the most prevalent danger signs (2%), and had high sensitivity (75%) which corresponds to other studies, which ranged from 45 to 100% [14, 23, 56]. The CHWs assessed breastfeeding and provided necessary instruction to the mother to feed the baby after the assessment. Though the CHWs assessed breastfeeding, the male physician did not because of cultural sensitivity.

Due to an inadequate number of newborns with tachypnea, we could not report the sensitivity and specificity of tachypnea in comparison to physician assessment.

One of the limitations of this study is the sample size was small compared to other studies discussed above. It has been suggested that to optimize validation study design, healthy and sick newborn infants should be equal in number [14]. However, in our study only 19 newborns (12.8%) were referred by the CHWs, and 15 (10.1%) were referred by the physician. Our study tried to look at the CHWs’ performance in the case of premature babies (*n* = 26). However, we did not have any newborn with birth weight < 2000 g, so we could not comment on CHWs’ performance on very low birth weight babies. Future studies can focus on larger group validation in very low birth weight babies.

## Conclusion

m-Health based, CHW-led assessment of newborns can be a potential strategy to reduce neonatal mortality and morbidity in underdeveloped and developing country settings. This study corroborates that mobile-health based assessment after five days of training and skill development can effectively extend the capabilities of community health workers to reach the most vulnerable newborns in rural areas that are less likely to receive postnatal care. Increasing access to quality postnatal care has been shown to reduce neonatal mortality. mHealth application training can potentially eliminate the need of long course extensive training of CHWs in providing new born care and barriers towards scaling up. CHWs can provide basic healthcare at the doorsteps in resource-poor communities efficiently if monitored and supervised well.

## Data Availability

Institutional Review Board approval was obtained for public sharing and presentation of data on a group level only. To maintain participants’ anonymity and confidentiality, the data set generated during the study will not be shared.

## Abbreviations

LMIC: Low- and Middle-Income Countries,
CHW: Community Health Worker
ANC: Antenatal Care
PNC: Postnatal care
AAP: American Academy of Pediatrics
UHC: Upazilla Health Complex (Sub-district hospital)
NGO: Non-Government Organization
TcB: Transcutaneous bilirubin
